# Bacteriophages as Weapons Against Bacterial Biofilms in the Food Industry

**DOI:** 10.3389/fmicb.2016.00825

**Published:** 2016-06-08

**Authors:** Diana Gutiérrez, Lorena Rodríguez-Rubio, Beatriz Martínez, Ana Rodríguez, Pilar García

**Affiliations:** ^1^Instituto de Productos Lácteos de Asturias, Consejo Superior de Investigaciones CientíficasVillaviciosa, Spain; ^2^Laboratory of Gene Technology, Katholieke Universiteit LeuvenLeuven, Belgium

**Keywords:** biofilm, bacteriophage, phage lytic proteins, food industry, disinfection

## Abstract

Microbiological contamination in the food industry is often attributed to the presence of biofilms in processing plants. Bacterial biofilms are complex communities of bacteria attached to a surface and surrounded by an extracellular polymeric material. Their extreme resistance to cleaning and disinfecting processes is related to a unique organization, which implies a differential bacterial growth and gene expression inside the biofilm. The impact of biofilms on health, and the economic consequences, has promoted the development of different approaches to control or remove biofilm formation. Recently, successful results in phage therapy have boosted new research in bacteriophages and phage lytic proteins for biofilm eradication. In this regard, this review examines the environmental factors that determine biofilm development in food-processing equipment. In addition, future perspectives for the use of bacteriophage-derived tools as disinfectants are discussed.

## Introduction

Food safety is an important issue for health authorities and industries due to the health impact and economic losses caused by the contamination of foodstuffs. Despite the implementation of Good Manufacturing Practices (GMP) and Hazard Analysis Critical Control Point (HACCPs) in food industries, in 2014 the European Food Safety Authority (EFSA) reported a total of 5,251 foodborne outbreaks resulting in 6,438 hospitalizations (EFSA and ECDC, 2016). In the United States, 866 foodborne outbreaks were reported in 2014, resulting in 714 hospitalizations: (http://wwwn.cdc.gov/foodborneoutbreaks/; accessed: November 27, 2015).

Food is often contaminated during processing and packaging through contact with equipment surfaces. Of note, contamination with hemolytic bacteria (*Staphylococcus aureus* and *Streptococcus agalactiae*) was detected in hands, hand-contact and food-contact surfaces in foodservice settings (DeVita et al., 2007); the presence of coliforms in washing water and industrial facilities are involved in the low microbiological quality of tomatoes (van Dyk et al., 2016) or the notable incidence of *S. aureus* and other pathogenic bacteria on food industry surfaces in Spain (Gutiérrez et al., 2012a) are some of the great number of reported examples.

In fact, elimination of bacteria in food processing environments is greatly hindered by the presence of biofilms which provide a reservoir of foodborne pathogens. Usually most bacteria are organized in multispecies communities attached to a surface as biofilms, which confer ecological advantages that free-living bacteria in planktonic cultures do not have. Extracellular matrix, composed of a mixture of polymeric compounds such as polysaccharides, proteins, nucleic acids, and lipids, keeps the bacteria in close proximity each other and forms channels to distribute water, nutrients, oxygen, enzymes, and cell debris. This structure provides a microenvironment with physicochemical gradients, horizontal gene transfer, and inter-cell communication. In addition, biofilm matrix protects the involved bacteria from environmental damages, antimicrobial agents, and host immune defenses (Flemming and Wingender, 2010). The low diffusion of antimicrobial substances through the matrix, together with an altered growth rate of bacteria constitutes the main barrier in the fight against relevant microorganisms living in biofilms (Donlan and Costerton, 2002).

Biofilm formation has notable implications in industrial processes, in particular in food processing, with a negative impact on food safety and the subsequent economic losses (Van Houdt and Michiels, 2010). In this regard, further studies about biofilm development and disassembly have been performed for important pathogenic bacteria such as *S. aureus* (Boles and Horswill, 2011; Periasamy et al., 2012) and *Listeria monocytogenes* (da Silva and De Martinis, 2013). Numerous biofilm control strategies have been proposed but the problem remains unsolved, probably because of the complexity of these structures, which contain both cells and extracellular substances. Ideally, a biofilm removal system should be able to get inside the biofilm structure and eliminate efficiently all the matrix components and the bacteria.

New approaches are focused on preventing biofilm formation by the development of anti-adhesive surfaces (Gao et al., 2011; Kesel et al., 2014; Salwiczek et al., 2014) or by the inhibition or reduction of bacterial adhesion (Cegelski et al., 2009; Pimentel-Filho et al., 2014). Moreover, removal strategies like physical and chemical treatments (Van Houdt and Michiels, 2010), antimicrobial photodynamic therapy (Sharma et al., 2011), induction of biofilm detachment (Cerca et al., 2013), blocking of biofilm regulation (Romling and Balsalobre, 2012), matrix degradation (Ramasubbu et al., 2005; Alkawash et al., 2006), and quorum sensing inhibitors (Hentzer et al., 2003) have been explored.

Another promising approach to control and eradicate biofilms is the use of bacteriophages. These viruses are harmless to humans, animals, and plants because they specifically target and kill bacteria. Virulent phages follow a lytic cycle where they multiply within bacteria to finally release the phage progeny by lysis of the cell. This process confers phages their antimicrobial activity. Phages have been used as treatment against human infections in countries from Eastern Europe, but the increase in antibiotic resistance has boosted new research and a notable interest worldwide for the use of phages to fight against pathogenic bacteria in clinical, veterinary, food safety, and environment (O’Flaherty et al., 2009; García et al., 2010). Phage-encoded lytic proteins such as endolysins and virion-associated peptidoglycan hydrolases (VAPGHs) have also been assessed as antimicrobial agents against pathogens (Schmelcher et al., 2012; Rodríguez-Rubio et al., 2013) and other phage-encoded proteins with polysaccharide depolymerase activity can be used as anti-biofilm agents (Cornelissen et al., 2011; Gutiérrez et al., 2012b, 2015a). Therefore, bacteriophages are not only bacterial killers but also a source of antimicrobial phage-derived proteins that can be exploited to fight against pathogenic bacteria.

Overall, the aim of this review is to assess both bacteriophages and bacteriophage-derived proteins as potential compounds to be applied as part of the cleaning and disinfecting processes of food-contact surfaces in the food industry.

## Relevance of Biofilms in the Food Industry and Disinfection Hurdles

Biofilm formation is a major concern in industrial settings, since it is one of the causes of operating troubles by decreasing heat transfer, blocking tubes, plugging filters, and causing damage to surfaces (Myszka and Czaczyk, 2011). Specifically in the food industry, the ability of bacteria to attach to food-contact surfaces provides a reservoir of contamination for pathogens with the consequent risks to human health. Analysis of the microbial composition of biofilms formed on food industrial surfaces revealed the presence of mixed biofilms including pathogenic and spoilage bacteria (Gounadaki et al., 2008; Gutiérrez et al., 2012a). These microorganisms can reach the food industry through several sources such as water, raw foods, animals, and can persist in the equipment for long periods of time. Therefore, food products can be contaminated at any stage of the food chain, even though all required cleaning protocols have been applied, because disinfecting and cleaning processes in the food industry are often ineffective. For instance, some microorganisms are able to survive after cleaning-in-place procedures, like in the case of dairy industries (Anand and Singh, 2013).

**FIGURE 1:**
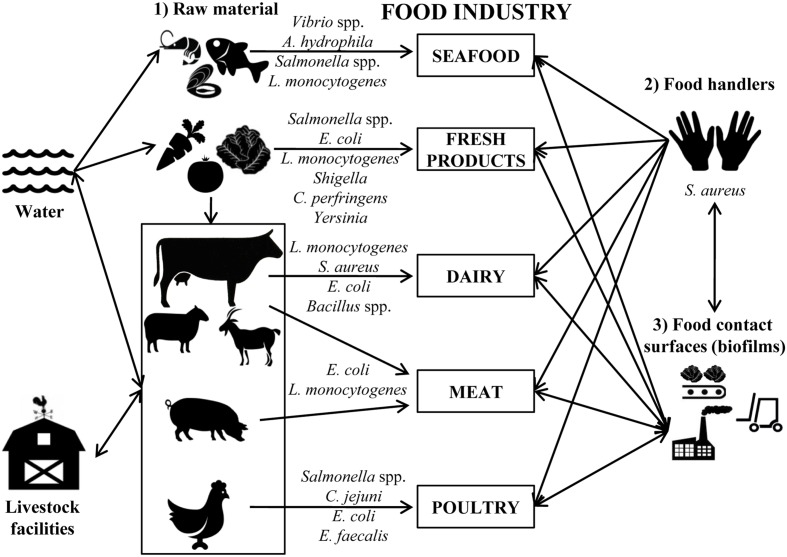
**Schematic representation of the main sources of contamination in food industries**. Most common bacteria detected in each agri-food industry are indicated.

Biofilms mainly cause problems in the dairy (Latorre et al., 2010), meat (Giaouris et al., 2014), poultry (Silagyi et al., 2009), seafood (Thimothe et al., 2004), and vegetable processing industries (Liu et al., 2013). Depending on the food-processing industry, the type of bacteria and the route of access to foodstuffs differs (**Figure 1**).

In seafood industries, the most common bacterial pathogens that form biofilms are *Vibrio* spp., *Aeromonas hydrophila*, *Salmonella* spp., and *L. monocytogenes* (Mizan et al., 2015). *Vibrio parahaemolyticus* can form biofilms on different surfaces including the chitin of oysters, and this process is recognized as vital to the physiology of these microorganisms (Thompson et al., 2010). *Vibrio cholerae* can form biofilms attached to the surface of phytoplankton and zooplankton, from where they can contaminate seafood products after consumption (Mizan et al., 2015). A correlation between the persistence of *Salmonella* spp. in the fish-processing industry and the ability for biofilm formation was also reported (Vestby et al., 2009). It was also demonstrated that other bacteria such as *L. monocytogenes* isolated from seafood industries can form biofilms on stainless steel surfaces (Gudmundsdottir et al., 2006).

In the fresh produce industry, bacteria such as *Salmonella*, *E. coli* O157:H7, *L. monocytogenes*, *Shigella*, *Bacillus cereus*, *Clostridium perfringens*, and *Yersinia* go into the processing facilities adhered to the plant tissues where they can grow forming biofilms (Beuchat, 2002; Da Silva Felicio et al., 2015). The accessibility of sanitizers to these microorganisms is hindered, not only by the presence of biofilms, but also by the intrinsic structure of vegetables, making it necessary to optimize the decontamination methods to extend their shelf-life (López-Gálvez et al., 2010).

In the dairy industry, most contamination comes from inadequate cleaning of the equipment and the presence of pathogenic bacteria; e.g., *L. monocytogenes* in milking equipment was determined to be the cause of contamination of bulk tank milk (Latorre et al., 2010). In addition, biofilm formation by *L. monocytogenes* may be promoted by specific conditions in the dairy industry like those used in cheese manufacturing (low pH values during milk fermentation and increased salt concentration). Thus, some strains increased their adherence to polystyrene after salt adaptation, and the exposure to acid increased the survival of cells adhering to stainless steel (Adriao et al., 2008). Milk proteins are also able to increase the attachment of *E. coli*, *L. monocytogenes*, and *S. aureus* to stainless steel (Barnes et al., 1999). On the other hand, members of the *Bacillus* genus are very common in dairy plants, where biofilm formation is triggered during milk lipolysis (Pasvolsky et al., 2014).

*E. coli* O157:H7 is a pathogenic bacteria also related with contamination in the meat industry. The ability of this bacterium to attach to meat-contact surfaces is influenced by the type of meat residues and the temperature. In fact, this microorganism significantly increases its counts number during inactivity periods of facilities (15°C) and also during cold storage temperatures (4°C; Dourou et al., 2011). Recently, it has been reported that *E. coli* O157:H7 strains isolated from a “high event period” (period of time during which commercial meat plants undergo a higher rate of contamination with this pathogen than normal) have a significantly higher potential of mature biofilm formation after incubation for 4–6 days, and also exhibit significantly stronger resistance to sanitization (Wang et al., 2014). *L. monocytogenes* was also isolated from bovine carcasses and meat processing facilities (Peccio et al., 2003; von Laer et al., 2009). The ability of this bacterium to colonize materials used in food processing surfaces (Rodriguez et al., 2008; Hingston et al., 2013), and to survive in niches that are difficult to sanitize such as countertops, cutting blades, or joints is well known (Verran et al., 2008).

*Salmonella* spp. and *Campylobacter* spp. are the most common pathogens found in poultry industries. *Salmonella* adhesion is influenced by different physicochemical properties of surfaces; for instance, *Salmonella* is able to grow at 16°C on stainless steel, while adherence was hindered on glass (De Oliveira et al., 2014). Recent studies have found that chicken meat exudation increases *Campylobacter jejuni* biofilm formation on glass, polystyrene, and stainless steel surfaces by covering and conditioning the surface (Brown et al., 2014). In addition, aerobic or stressful conditions (Reuter et al., 2010) and the presence of other bacteria such as *Enterococcus faecalis* and *Staphylococcus simulans*, also found in poultry processing environments, increase the level of biofilm formation allowing the survival of *C. jejuni* under detrimental conditions (Teh et al., 2010).

Overall, the main concern about biofilms is their wide resistance to disinfectants commonly used in food industries, which include quaternary ammonium compounds such as benzalkonium chloride (BAC). The resistance to these compounds shown by several foodborne pathogens results in their reduced efficacy (Saa-Ibusquiza et al., 2011); e.g., *L. monocytogenes* is able to modify the physicochemical properties of the cell surface as a response to low concentrations of BAC resulting in a higher resistance to this compound (Bisbiroulas et al., 2011). Biofilm resistance to antimicrobials is attributed to several intrinsic biofilm properties such as reduced diffusion, physiological changes of cells, reduced growth rates, and the production of enzymes that degrade the antimicrobial compounds (Bridier et al., 2011). In this regard, it has been shown that the extracellular material constitutes a physical barrier for biocides and the chemical interaction with this material reduces the rate of diffusion to the biofilm inside. Besides the physical barrier found by the antimicrobial compounds to penetrate into the biofilm, there is a physiological resistance due to the altered growth rate of cells forming the biofilm, which grow more slowly than planktonic cells and consequently are less affected by the biocide (Evans et al., 1991). The presence of persister cells, which are tolerant to antimicrobials, could also explain the resistance of biofilm to biocides along with an adaptive tolerance (Simoes et al., 2011). Thus, it has been suggested that exposure to sublethal concentrations of biocides allows bacterial adaptation and survival at the level of biocide concentrations used in the food environment (Capita et al., 2014). In many bacteria, such as *S. aureus*, multidrug eﬄux pumps are responsible for this biocide resistance (Rouch et al., 1990). In fact, prolonged exposure to sublethal concentrations of biocides can lead to the overexpression of these eﬄux pumps and hence to the increased multidrug resistance in bacteria (Gilbert and McBain, 2003). In this regard, *in vitro* cross-resistance with antibiotics has been described for some biocide-resistant foodborne pathogens (Davin-Regli and Pages, 2012; Gnanadhas et al., 2013) supporting the need for monitoring and regulating the usage of biocides. The maturation stage of biofilms may also enhance resistance to disinfectants, since it has been reported that sodium hypochlorite, sodium hydroxide, and BAC failed to eradicate mature *Salmonella* biofilms (Corcoran et al., 2014).

## Impact of Food-Processing Conditions on Biofilm Development

**FIGURE 2:**
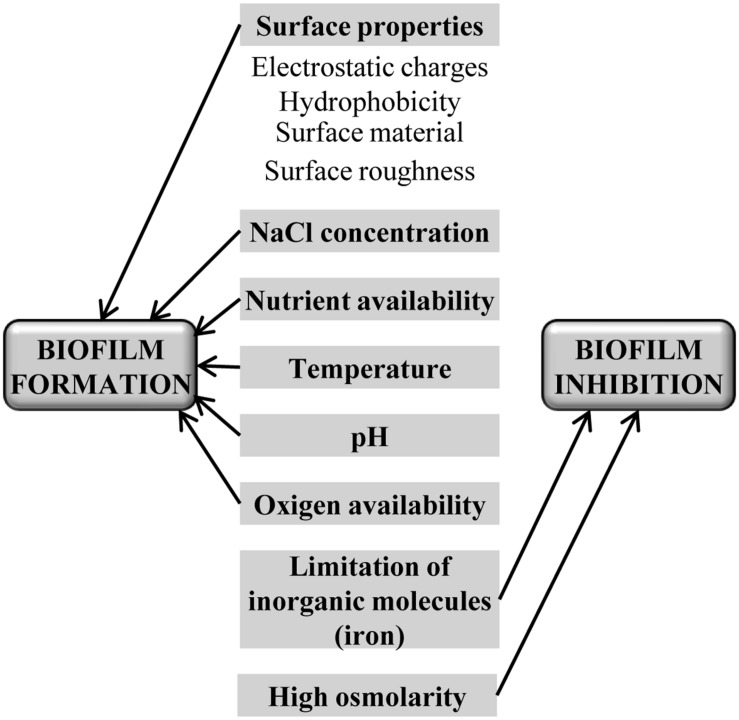
**Main food industry’s parameters that can influence biofilm development**.

In food processing environments, there are a number of variable conditions such as temperature, pH, oxygen and nutrients availability, and surface type, which can modulate biofilm development (**Figure 2**). Surface properties such as electrostatic charges, hydrophobicity, and roughness influence biofilm development in some species. For instance, hydrophilic surfaces are more quickly colonized by *L. monocytogenes* (Chavant et al., 2002), whereas *S. aureus* have not shown any differences between hydrophobic and hydrophilic surfaces (da Silva Meira et al., 2012), and *Salmonella* has a higher ability to adhere to some materials used in food-contact surfaces like Teflon, followed by stainless steel, glass, Buna-N rubber, and polyurethane (Chia et al., 2009). In some cases, biofilm retention is more affected by the surface roughness than by the chemical composition (Tang et al., 2011). Other components of food environments such as NaCl also contribute to increase the adhesion of *L. monocytogenes* to surfaces (Jensen et al., 2007), although it is influenced by temperature and nutrients as well (Moltz and Martin, 2005), and even by the presence of other bacteria in the food-processing environment (Carpentier and Chassaing, 2004).

Food-related environmental factors have a variable impact on biofilm development. Bacteria sense these factors through sophisticated intracellular and extracellular signaling networks resulting in a negative or positive response (Karatan and Watnick, 2009). For instance, nutrient limitation induces *Salmonella enterica* serovar Typhimurium to biofilm formation (Gerstel and Romling, 2001), whereas *V. cholerae* needs a nutrient-rich environment to develop a biofilm structure (Yildiz et al., 2004). Similarly, in *S. aureus* an increase in biofilm formation was observed in a nutrient-rich growth media (Herrera et al., 2007) and at high incubation temperatures (Vázquez-Sánchez et al., 2013). Secondary metabolites such as antibiotics may also induce biofilm formation (Hoffman et al., 2005). Another example was recently reported by Nesse et al. (2014), where potentially human-pathogenic *E. coli* from the ovine reservoir can form biofilms under conditions used in the food production chain [on different surfaces such as stainless steel, glass, and polystyrene and at temperatures relevant for food production and handling (12, 20, and 37°C)]. Of note, for most bacteria, limitation of inorganic molecules such as iron and inorganic phosphate has an inhibitory effect on biofilm formation (Mey et al., 2005; Monds et al., 2007), and high osmolarity inhibits in general, biofilm formation, although this effect is clearly dependent on the osmolyte (Jubelin et al., 2005).

## Bacteriophages Properties as Antimicrobials

Bacteriophages are viruses of prokaryotes widespread in all habitats where their hosts are located. Classifications of bacteriophages are based on their shape, size, and kind of nucleic acid. The most abundant belong to the *Caudovirales* order (tailed-bacteriophages), which is divided into three families (*Myoviridae*, *Podoviridae*, and *Siphoviridae*) according to the microscopic features of the tail morphology. Bacteriophages belonging to the *Siphoviridae* family are the most abundant (57.3%; Ackermann and Prangishvili, 2012).

Bacteriophages can infect bacteria by following two different life cycles, lytic and lysogenic (Kutter et al., 2004). In most phages, the lytic cycle ends with the lysis of host bacteria and the progeny release. Thus, antimicrobial properties of bacteriophages are linked to the lytic cycle (lytic phages) since the infected host is intended to die. On the contrary, the lysogenic cycle followed by temperate bacteriophages implies the survival and establishment of the phage genome into the bacterial chromosome (prophage) until environmental signals trigger a lytic cycle, thereby killing only a part of the infected population. In addition, lysogenic bacteria, carrying a prophage, are resistant to infection for a related phage (superinfection immunity; Kutter et al., 2004).

**FIGURE 3:**
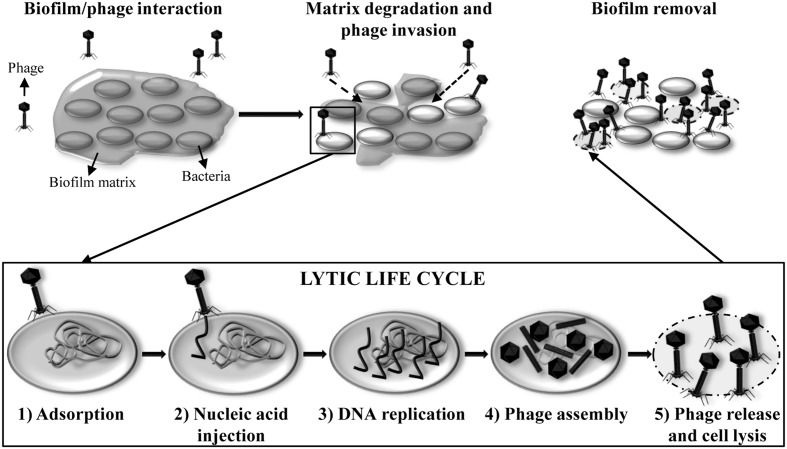
**Lytic life cycle of phages inside a biofilm. (1)** Adsorption of the phage particle onto the host bacterial cell surface. Tail fibers bind to specific receptors on the cell surface. **(2)** Injection of the nucleic acid into the cytoplasm of the host bacterium. **(3)** Replication of the phage genome in multiple copies. Phage early genes are expressed to regulate the host metabolic machinery to be subjected to phage propagation. **(4)** Formation of new phage particles by expression of the phage late genes and assembly of the phage heads and tails, packaging of the nucleic acid inside the heads and maturation of the virions. **(5)** Lysis of the host bacterium and release of the new phage progeny ready to infect other cells in the biofilm and start a new cycle.

**FIGURE 4:**
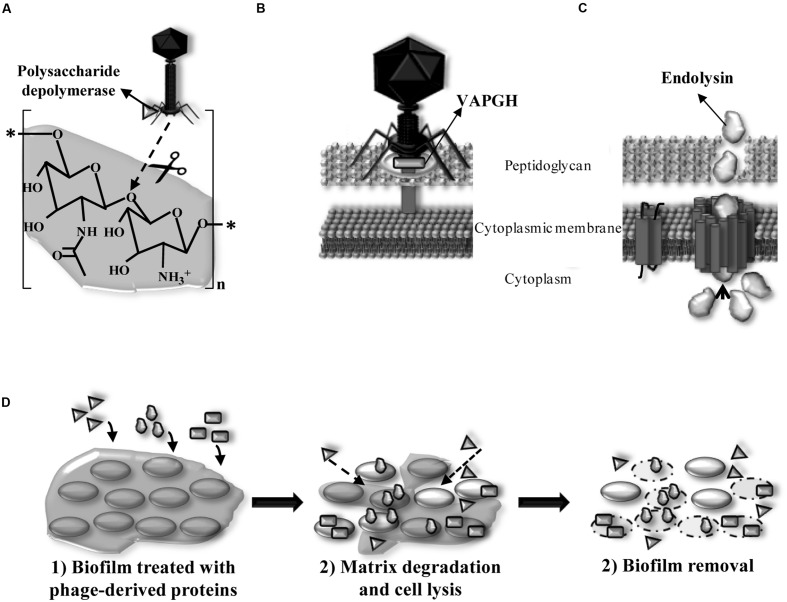
**(A)** Location of exopolysaccharide depolymerase degrading β-(1,6) bonds of the biofilm extracellular matrix (PIA/PNAG) of staphylococcal species in the phage particle and mode of action. **(B)** Location of virion-associated peptidoglycan hydrolase (VAPGH) at the phage particle and its role in the infection process. **(C)** Structure of Gram-positive bacteria cell wall and role of the endolysin during the bacterial lysis. **(D)** Activity of phage derived proteins when added exogenously and their application as anti-biofilm agents degrading polysaccharidic matrices (polysaccharide depolymerases) and lysing bacteria (VAPGHs and endolysins).

In nature, most bacteria are living in biofilms (Hall-Stoodley et al., 2004). The interaction between the host bacteria and the lytic phages occurs in six different steps (**Figure 3**). The adsorption of the bacteriophage and release of the new phage progeny play a key role in the bacteriophage infection process. When host bacteria are included in a biofilm, the biofilm matrix can constitute a first physical barrier to the phage. To solve this problem, some phages possess polysaccharide depolymerases which are specific hydrolytic enzymes that can use polysaccharides or polysaccharides derivatives as substrate (Pires et al., 2016; **Figure 4A**). Numerous studies have shown that polysaccharide depolymerase activity is related to tail-spike proteins which are components of the tail of many bacteriophages (Barbirz et al., 2009). The presence of polysaccharide depolymerases confers the phage an important advantage since it enhances the process of invasion and dispersion through the biofilm to start the infection process of new bacteria. Moreover, some phages are provided with lytic enzymes which are named VAPGHs, with a role in the first step of the infection cycle (**Figure 4B**). Their activity produces a small hole in the cell wall through which phage genetic material reaches the cytoplasm, being responsible for the “lysis from without” caused by the adsorption of a high number of phages to the cell at the initial infection step (Moak and Molineux, 2004). Recently, these proteins have also been proposed as new antimicrobials due to their lytic activity (Rodríguez-Rubio et al., 2013).

Double-stranded phages encode lytic proteins, named endolysins, which act along with holins to disrupt the cell wall and lyse the host bacteria at the last step of the lytic infection cycle (**Figure 4C**). Endolysins, which are also peptidoglycan hydrolases, access the periplasmic space through holes formed by holins in the cytoplasmic membrane. Hydrolysis of covalent bonds in the peptidoglycan molecule produces the destabilization of the cell wall structure and lysis by the increase of the osmotic pressure inside the cytoplasm. In Gram-positive bacteria, endolysins are able to degrade the peptidoglycan when they are added from outside the cell, which gives them an antimicrobial activity (enzybiotics; Fischetti, 2008). In Gram-negative bacteria, peptidoglycan is protected by the outer membrane, these bacteria being insensitive to endolysins. Nowadays, research efforts made into endolysin applications against Gram-negative pathogens are changing this rule. This is the case of Artilysins that combine a polycationic peptide able to penetrate the outer membrane with an endolysin, which renders a protein with high bactericidal activity against Gram-negative pathogens (Briers et al., 2014a,b).

The use of phage-encoded proteins as antimicrobials has some advantages over the use of the viral particles; e.g., no resistant bacteria to phage lytic proteins has been described to date (Nelson et al., 2012; Rodríguez-Rubio et al., 2013). Additionally, the spectrum of activity of endolysins is usually broader than the host range of bacteriophages and, there are no risks of transferring virulence genes (Fischetti, 2008).

Due to the above described working mechanisms, bacteriophages and phage-derived proteins could be used in the production of foodstuffs against unwanted bacteria to ensure quality, safety, and good hygienic conditions, covering the entire food chain production (“from farm to fork”). This includes strategies to improve animal health (phage therapy), decontamination of fresh-food and ready-to-eat products, disinfection of food-contact surfaces, as well as their use as biopreservatives to inhibit the development of pathogenic or spoilage bacteria during storage, and also as tools for detecting undesirable bacteria through the different manufacturing steps (García et al., 2010).

## Control of Biofilms Using Bacteriophages and Phage-Derived Proteins

The recent interest in phage therapy as an alternative to conventional antibiotics has fostered the use of phages in multiple applications, among which their use as anti-biofilm agents should be noticed. Biofilms are the lifestyle of bacteria in nature and therefore, phages have evolved to infect cells at this stage (Hall-Stoodley et al., 2004). There are two putative limitations for phage infection of cells inside the biofilm. First, the accessibility of phages to cells due to the structure of biofilm and the presence of extracellular material. Briandet et al. (2008) demonstrated that phage c2 was able to penetrate inside the *Lactococcus lactis* biofilm structure through water channels and cell clusters; in addition, the infection of *E. coli* surface-attached cells was confirmed by using T4 fluorescently labeled phages (Doolittle et al., 1996). Some phages, provided with exopolysaccharide depolymerases, can degrade the extracellular polymeric material, thus facilitating the entrance of phages into the deeper layers of the biofilms with subsequent lysis of the target bacteria (Parasion et al., 2014). The second limitation in the treatment of biofilms with bacteriophages is the metabolic status of a proportion of the population, persister cells and stationary phase bacteria, which have a slow metabolism. Bacteriophages infect preferably exponentially growing bacteria but recently, it has been demonstrated that persister bacteria can be infected by phages when bacteria switch to normal growth rate (Pearl et al., 2008). Moreover, persister cells can be removed by phage lytic proteins (Gutiérrez et al., 2014) due to these proteins are able to easily penetrate into the biofilms (Shen et al., 2013). Furthermore, bacteriophages can be engineered to express proteins intended to enhance their anti-biofilm properties. For instance, phage T7 was genetically engineered to incorporate the gene *dspB* encoding a polysaccharide depolymerase from *Actinobacillus actinomycetemcomitans*, which was more effective at reducing the bacterial count in *E. coli* biofilms (Lu and Collins, 2007). In addition, it has been demonstrated that engineered bacteriophages overexpressing proteins able to suppress bacterial SOS response network in *E. coli* are more effective against persister cells (Lu and Collins, 2009).

Several studies using biofilms preformed in laboratory conditions confirm the potential of phages in biofilm removal (**Table 1**). For biofilms formed by pathogenic bacteria with relevance in the food industry, there is evidence of effective removal in different conditions and using materials similar to those found in food-contact surfaces. Regarding this, three phages LiMN4L, LiMN4p, and LiMN17 infecting *L. monocytogenes* were assayed, individually and as a cocktail, against 7-day biofilms formed by a mixture of three strains on stainless steel coupons (10^4^ cfu/cm^2^), previously covered with a fish broth layer that simulated seafood processing facilities. Treatments with the single phages (10^9^ pfu/ml) reduced adhered bacterial cells to up 3 log units, whereas treatment with the phage cocktail reduced cell counts to undetectable levels after 75 min (Ganegama-Arachchi et al., 2013). Similarly, a treatment with phage P100 (10^9^ pfu/ml) reduced biofilms formed by *L. monocytogenes* strains in 3.5–5.4 log/cm^2^, irrespective of the serotype, growth conditions and biofilm level (Soni and Nannapaneni, 2010). Despite the efficacy of bacteriophages to reduce *L. monocytogenes* biofilms, there is evidence that complete removal is not always achieved. By using epifluorescence microscopy, *L. monocytogenes* was monitored after treatment with phage P100 (10^8^ pfu/ml) and, although disaggregation of biofilms could be observed after 8 h, viable cells were still present up to 48 h later, indicating that other sanitization methodologies should be used in combination with phages (Montañez-Izquierdo et al., 2012).

**Table 1:** Application of bacteriophages and phage proteins for biofilm removal.

Phage or phage protein	Scope of application	Bacteria	Efficacy of the treatment	Reference
Phages LiMN4L, LiMN4p, and LiMN17	Stainless steel	*L. monocytogenes*	Phage cocktail reduced biofilm cell counts to undetectable levels after 75 min	Ganegama-Arachchi et al., 2013
Phage P100	Stainless steel	*L. monocytogenes*	Reduction in the cell counts from 3.5 to 5.4 log units/cm^2^	Soni and Nannapaneni, 2010
Phage P100	Stainless steel	*L. monocytogenes*	Reduction of the biofilm cell counts to undetectable levels after 48 h	Montañez-Izquierdo et al., 2012
Phage K and phage derivatives	Polystyrene	*S. aureus*	Complete elimination of the biomass after 72 h of incubation. Complete inhibition of biofilm formation was achieved when co-culturing phage mixture and bacteria	Kelly et al., 2012
Phage K and DRA88	Polystyrene	*S. aureus*	Complete elimination of the biomass after 48 h of treatment	Alves et al., 2014
Phages ISP, Romulus, and Remus	Polystyrene	*S. aureus*	Biofilm reduction of 37.8, 34.4, and 60.4% after 24 h treatment when using phages ISP, Romulus, and Remus, respectively	Vandersteegen et al., 2013
Phages phiIPLA-RODI and phiIPLA-C1C	Polystyrene	*S. aureus*	Reduction by 2 log units/well was achieved after 8 h of treatment	Gutiérrez et al., 2015b
Phage SAP-26	Polystyrene	*S. aureus*	Reduction of bacteria about 28% after phage treatment, while a synergistic effect with rifampicin allows a reduction of about 65%	Rahman et al., 2011
Phage CP8 and CP30	Glass	*C. jejuni*	Reduction in the biofilm cell counts of 1–3 log units/cm^2^	Siringan et al., 2011
Phage KH1	Stainless steel	*E. coli* O157:H7	Reduction of 1.2 log units per coupon after 4 days treatment at 4°C	Sharma et al., 2005
BEC8 (phage mixture)	Stainless steel, ceramic tile, and high density polyethylene	*E. coli* O157:H7	Reduction of the biofilm cell counts to undetectable levels after 1 h of treatment at 37, 23, and 12°C	Viazis et al., 2011
Phage mixture	Spinach harvester blade	*E. coli* O157:H7	Reduction of biofilm cell counts by 4.5 log units per blade after 2 h of treatment	Patel et al., 2011
Phage T4	Polystyrene	*E. coli* O157:H7	Complete elimination of the biomass after phage treatment combined with cefotaxime	Ryan et al., 2012
Endolysin from phage phi11	Polystyrene	*S. aureus*	Complete elimination of the biomass after 2 h of treatment at 37°C	Sass and Bierbaum, 2007
Endolysin SAL-2	Polystyrene	*S. aureus*	Reduction of the biomass after 2 h of treatment at 37°C	Son et al., 2010
Endolysin LysH5	Polystyrene	*S. aureus*	Reduction of biofilm cell counts by 1–3 log units after 3 h of treatment	Gutiérrez et al., 2014
Domain CHAP_K_ derived from endolysin LysK	Polystyrene	*S. aureus*	Complete elimination of the biomass after 4 h of incubation Complete inhibition of biofilm formation was achieved	Fenton et al., 2013
Chimeric lysin ClyH	Polystyrene	*S. aureus*	Reduction of the biomass in more than 60% after 30 min of treatment	Yang et al., 2014
Endolysin Lys68	Polystyrene	*S.* Typhimurium	Reduction of biofilm cell counts by 1 log unit after 2 h of treatment in the presence of outer membrane permeabilizers	Oliveira et al., 2014
Exopolysaccharide depolymerase Dpo7	Polystyrene	*S. aureus*	Degradation of 30% of the polysaccharidic matrix of the biofilm	Gutiérrez et al., 2015a

*Staphylococcus aureus* is another important foodborne pathogen with the ability to form biofilms on different surface’s materials. Staphylococcal phage K and a mixture of derivative phages with broader host ranges were used to effectively prevent *S. aureus* biofilm formation over incubation periods of 48 h. It was also shown that the removal of bacteria by the phage cocktail (10^9^ pfu/ml) was time-dependent, with the highest reduction occurring after 72 h at 37°C (Kelly et al., 2012). A similar result was obtained using phage K combined with another staphylococcal phage, DRA88 (MOI 10), to treat established biofilms produced by three *S. aureus* isolates, which were significantly reduced after 4 h and completely removed after 48 h at 37°C (Alves et al., 2014). Other staphylococcal phages such as ISP, Romulus, and Remus applied individually at 10^9^ phages per polystyrene peg were able to degrade by 37.8, 34.4, and 60.4%, respectively, an *S. aureus* PS47 biofilm after 24 h (Vandersteegen et al., 2013). Similar results were obtained after the application of phages phiIPLA-RODI, phiIPLA-C1C, and a mixture of both phages, against biofilms formed by *S. aureus* where a reduction by about 2 log units was achieved after 8 h of treatment at 37°C (Gutiérrez et al., 2015b). In some cases, however, it was also necessary to combine phages with other antimicrobials to increase their effectiveness. Thus, treatment of 1-day-old biofilms formed by *S. aureus* D43 strain with phage SAP-26 reduced live bacteria by about 28% while a synergistic effect with rifampicin allowed a reduction of about 65% (Rahman et al., 2011).

Bacteriophages were also assayed against *C. jejuni* biofilms. Two virulent phages, CP8 and CP30 led to 1–3 log cfu/cm^2^ reduction in viable counts after 24 h of treatment. However, a high percentage of bacteriophage-resistant bacteria in biofilms were observed for some *C. jejuni* strains (Siringan et al., 2011).

Sharma et al. (2005) assayed the lytic bacteriophage KH1 (7.7 log pfu/ml) against stainless steel coupons containing *E. coli* O157:H7 biofilms (2.6 log cfu/coupon). These were treated for 4 days at 4°C and a reduction of 1.2 log units per coupon was observed. Better results were obtained when treating *E. coli* O157:H7 biofilms preformed on other materials typically used in food processing surfaces (stainless steel, ceramic tile, and high density polyethylene), since a reduction to undetectable levels was observed after 1 h of treatment at 23°C with a phage mixture named BEC8 (MOI 100; Viazis et al., 2011). The use of a phage mixture to remove biofilms formed on blades used to harvest spinach was also demonstrated, a reduction of 4.5 log units of the viable cells of *E. coli* O157:H7 being achieved after 2 h of phage treatment (Patel et al., 2011). As it was previously reported, a combination of T4 bacteriophage and cefotaxime significantly enhanced the eradication of *E. coli* biofilms when compared to treatment with phage alone (Ryan et al., 2012).

Phage lytic proteins are also an alternative for removing bacterial biofilms in food-related environments (**Figure 4D**; **Table 1**). Endolysin from phage phi11 (10 μg/well) was able to remove biofilms formed by *S. aureus* strains on polystyrene surfaces after 2 h at 37°C (Sass and Bierbaum, 2007). Similarly, endolysin SAL-2 from bacteriophage SAP-2 eliminated *S. aureus* biofilms using 15 μg/well (Son et al., 2010). Recently, Gutiérrez et al. (2014) showed that endolysin LysH5 (0.15 μM) is able to remove staphylococcal biofilms after treatment of 12 h at 37°C and even to lyse persister cells. Engineered endolysins, by deletion or shuﬄing domains, have also been successfully used as anti-biofilm agents. For instance, peptidase CHAP_k_ (31.25 μg/ml), derived from the staphylococcal endolysin LysK, was able to completely prevent biofilm formation. This protein also removed staphylococcal biofilms after treatment of 4 h at 37°C (Fenton et al., 2013). In addition, the minimum concentration (6.2–50 mg/l) of ClyH, a staphylococcal chimeric lysin, required for *S. aureus* biofilm eradication was lower than that needed when antibiotics were used (Yang et al., 2014). This protein contains the catalytic domain of endolysin Ply187 and the cell wall binding domain of phiNM3 lysin. Regarding biofilms formed by Gram-negative bacteria, removal of these structures by using endolysins needs an additional component to disestablish the outer membrane. Biofilms formed by *S. enterica* serovar Typhimurium were treated with endolysin Lys68 (2 μM), which reduced by 1 log unit the viable cells in preformed biofilms after 2 h of incubation in the presence of outer membrane permeabilizers (Oliveira et al., 2014).

Regarding phage-encoded exopolysaccharide depolymerases, there is scarce data about the biofilm dispersion mediated by these proteins but they seem to be very promising. Cornelissen et al. (2011) identified an exopolysaccharide-degrading activity associated to a tail spike protein from *Pseudomonas putida* phage Φ15, which is involved in the hydrolysis of extracellular material. However, the addition of the purified tail spike protein did not result in biofilm removal. Since the addition of 10^6^ phages yielded a significant biofilm degradation of 37% in 24 h, this seems to require phage amplification (Cornelissen et al., 2012). Recently, an exopolysaccharide depolymerase named Dpo7 was identified in the *S. epidermidis* phage phiIPLA7. Purified protein was used to treat *S. aureus* biofilms, showing its ability to degrade up to 30% of the polysaccharidic matrix formed by *S. aureus* 15981 (Gutiérrez et al., 2015a).

Overall, these results showed a noticeable potential of phages and phage-derived proteins, but undoubtedly additional studies are necessary to transfer this knowledge to the food industry. For instance, application of these anti-biofilm compounds would be feasible as long as their application can be implemented as part of the standard processes of cleaning in the industrial facilities. Therefore, the study of synergy/antagonism with disinfectants and the effectiveness at temperatures commonly used in the industry could be relevant. It should be also noticed the scarce data available about the use of phages and phage lytic proteins against mixed biofilms formed by different strains from several species in food industrial surfaces. This gap should be filled in to go further into the control of bacterial biofilms.

## Future Perspectives for Phage-Based Disinfectants

The most important issues to address before the implementation of phages and phage-derived proteins as disinfectants are the following.

### Safety

Beyond efficiency, safety of phage-based products must be a priority to take into account. Only phages fully characterized at molecular level and with the complete genome sequenced should be taken into consideration as potential components of disinfectants to avoid the presence of virulence and antibiotic resistance genes. These phages must be lytic, since temperate bacteriophages have the ability to integrate their genomes into their host bacterium’s chromosome, and non-transducers, i.e., without the ability to transfer genetic material from host bacteria. One of the most important characteristics of bacteriophages, their high specificity for the host bacteria, could be a potential limitation in their use as disinfectants. A cocktail of different phages with overlapping host ranges or the use of polyvalent phages with a wide host range would solve this problem. Finally, in the selection of phages to be included in the cocktail, the presence of those encoding polysaccharide depolymerase enzymes should be preferred.

Regarding the safety of engineered phages, the main hurdle for their use is the generalized opposition of consumers to genetic manipulation, despite of engineered phages can overcome the limitations of phages as antimicrobials and even specific modifications can eliminate some of their risks such as virulence genes or gene transfer (Nobrega et al., 2015).

Before the extensive use of bacteriophages as disinfectants, the absence of an ecological impact on the environment must be also guaranteed. In this regard, bacteriophages should be inactivated before their release outside the industry settings. Some commercial sanitizers and disinfectants commonly used in the food industry can be effective to inactivate phages, oxidizing agents and quaternary ammonium compounds being the most efficient ones (Campagna et al., 2014). Other treatments such as CO_2_, high pressure and UV light could be evaluated for each phage (Guglielmotti et al., 2011; Cheng et al., 2013). On the other hand, development of phage insensitive bacteria could be a cause of concern, since they may hamper the effectiveness of the phage disinfection process. Generally, the rate at which bacteria develop resistance is very low, especially when a cocktail of different phages is used. Moreover, phage-insensitive bacteria are associated with a reduced fitness (Gutiérrez et al., 2015b); therefore, this question is expected to have minor relevance.

In this context, endolysins have some important advantages compared to phages due to their proteinaceous nature, which is easily degraded in the environment. Regarding safety, the most important is their inability to transfer virulence genes.

### Large-Scale Production

Implementation of phages as disinfectants in the food industry implies obtaining large volumes of phage suspensions with high titer using an inexpensive protocol. Therefore, some work is still necessary to optimize propagation and purification processes for each phage (Bourdin et al., 2014). In this regard, phages should be propagated in a non-pathogenic bacterium and then purified in order to remove cell debris or other contaminating substances. For preparations of bacteriophages infecting Gram-negative bacteria some procedures to remove endotoxin have been reported (Boratynski et al., 2004), and several companies also sell kits for endotoxin detection. However, the importance of these contaminating components in medical applications is more crucial than for disinfection. In the latter, undesirable effects of phages could be related with allergy by skin contact or by inhalation of aerosols. At present, nevertheless, there are no reported side effects of the use of bacteriophages in animal models of phage therapy applications (Golkar et al., 2014), which is not surprising as phages are abundant in human microbiota (De Paepe et al., 2014) and in the environment (Díaz-Munoz and Koskella, 2014). Overall, the purification methods used at laboratory-scale are well defined and consist of the precipitation of phages by polyethylene glycol followed by purification of phages in a cesium chloride gradient. However, these procedures are neither easy to scale up, nor cheap for the large-scale production required for the application of phages as disinfectants. New purification alternatives are being studied, e.g., suitable methods designed for purification of bionanoparticles, based on anion-exchange chromatography, with a 60% recovery of viable phages (Adriaenssens et al., 2012). Alternatives to centrifugation such as tangential flow filtration and specific membrane materials could also be explored (Hambsch et al., 2012).

The main drawback in the extensive use of endolysins might be the difficulty of their effective expression in *E. coli* (Rosano and Ceccarelli, 2014). Other bacteria like *L. lactis* have been proposed as suitable cell factories (D’Souza et al., 2012), but even the expression might need to be optimized (Rodríguez-Rubio et al., 2012). Moreover, large-scale production and purification of proteins is a costly process in itself. A similar scenario might be drawn for exopolysaccharide depolymerases due to the requirement of having large amounts of pure protein. In addition, more research is necessary to find out how specific the activity is for its substrate.

Regarding phage-derived proteins, thermostability seems to be a challenge, and a big concern, when applying enzymes for disinfection. However, heat stability seems to be a recurring property of phage structural lysins or VAPGHs (Rodríguez-Rubio et al., 2013). On the other hand, although the thermolabile nature of endolysins is well known (Varea et al., 2004; Obeso et al., 2008; Filatova et al., 2010; Heselpoth and Nelson, 2012), there are exceptions to the rule. In fact, two novel thermostable endolysins have recently been described, Lys68 from *Salmonella* phage phi68 (Oliveira et al., 2014) and Ph2119 from bacteriophage Ph2119 infecting *Thermus scotoductus* strain MAT2119 (Plotka et al., 2014). This thermostability supports the potential use of these phage-derived enzymes as disinfectants.

In addition to propagation and purification of phages and phage-derived proteins, other parameters such as a proper formulation, stability under non-refrigerated conditions, and lytic activity under usual conditions for the food industry should also be studied. No data about phage formulations and storage other than lyophilization (Merabishvili et al., 2013) and spray drying (Vandenheuvel et al., 2013) is available. Survival of phages in both processes is strictly dependent on an appropriate protector, in most cases sucrose being the most effective agent to protect phages (Merabishvili et al., 2013). However, this sugar is not suitable as excipient for disinfection processes. The controlled delivery of phages and their stability in encapsulated microspheres are worth studying. In fact, strategies under development for medical applications include phage encapsulation using different materials suitable for oral delivery or inhalation (Puapermpoonsiri et al., 2009; Dini et al., 2012; Balcao et al., 2014). Furthermore, bacteriophages could be useful to develop specific antimicrobial packaging materials for use in the food industry (Han et al., 2014).

### Market and Regulatory

The potential of phages in the food industry is so extensive that several companies have developed phage-based products against important foodborne pathogens that could be used as disinfectants on surfaces and as food-processing aids. OmniLytics Inc. (Sandy, UT, USA) has developed two washing products, BacWash^TM^ against *Salmonella*, and Finalyse^TM^ against *E. coli* O157:H7, marketed by Elanco (Greenfield, IN, USA). Intralytix Inc. (Baltimore, MD, USA) developed three phage products, ListShield^TM^, EcoShield^TM^, and SalmoFresh^TM^, to be used in the food industry against *L. monocytogenes*, *E. coli*, and *Salmonella*, respectively. In Europe, Micreos BV (Wageningen, Netherlands) has commercialized Listex^TM^ (P100) against *L. monocytogenes*, and Salmonelex^TM^ against *Salmonella*. All these products are setting a precedent for future approval of phages as disinfectants. In fact, one of the most important drawbacks in the use of phage-based products might be the specific regulatory framework of each country. The US Department of Agriculture and FDA have already approved the use of several phage-based products, mentioned above, in food production environments, including their application as both food biopreservatives and disinfectants of food-contact surfaces. In Europe, however, the EFSA has argued that it is not clear whether bacteriophages can protect food against a re-contamination in spite of having been reported that bacteriophages are effective in the elimination of pathogens (EFSA, 2012). Finally, it is worth noticing that bacteriophages have also been approved as processing-aids in food processing and handling in several countries, but nothing has been reported about the use of phages as antimicrobial agents for the cleaning of industrial surfaces.

## Concluding Remarks

The development of new disinfection products, non-toxic to humans and friendly to the environment, has good prospects for the future. Bacteriophage-based disinfectants fulfill all the requirements regarding effectiveness and safety. However, two main challenges have to be overcome before the implementation of phages in the food industry: (i) more research is necessary to solve the technical problems in manufacturing, such as the scaling up of the processes of propagation or expression, and purification of phages and proteins, and (ii) a regulatory framework for phage applications should be established, which would boost investment in these new products.

## Author Contributions

PG, AR, and BM conceived the revision work. DG and LR-R designed the figures. DG, LR-R, BM, AR, and PG wrote the manuscript.

## Conflict of Interest Statement

The authors declare that the research was conducted in the absence of any commercial or financial relationships that could be construed as a potential conflict of interest.
